# *Vaccinium oldhamii* Fruit Inhibits Lipid Accumulation in 3T3-L1 Cells and Diet-Induced Obese Animals

**DOI:** 10.3390/nu17081346

**Published:** 2025-04-14

**Authors:** Young-Hyeon Lee, Mikyoung You, Hyeon-A Kim

**Affiliations:** 1 Department of Food and Nutrition, Mokpo National University, Muan-gun 58554, Republic of Korea; ciesl7@naver.com (Y.-H.L.); myou@mnu.ac.kr (M.Y.); 2 Convergence Center for Green Anti-Aging Research, Mokpo National University, Muan-gun 58554, Republic of Korea

**Keywords:** *Vaccinium oldhamii*, lipid accumulation, anti-obesity, AMPK

## Abstract

Background/Objectives: Obesity is a significant global health concern, and the natural bioactive compounds with anti-obesity effects remain challenging. This study aims to examine the anti-obesity effect and the potential mechanism of *Vaccinium oldhamii* fruit water extract (VOW). Methods: Lipid accumulation, AMP-activated protein kinase (AMPK) activity, and Wnt/β-catenin signaling were evaluated in 3T3-L1 cells. In high-fat and high-sucrose diet (HFHSD)-induced obese mice, body weight, food intake, fat weight, serum lipid profiles, and adipogenic transcription factors were assessed. The most effective VOW fraction was selected by Oil Red O (ORO) staining and its mechanism was studied in 3T3-L1 cells. Results: VOW treatment significantly inhibited cellular lipid accumulation and suppressed phosphorylation of AMPK and its downstream protein, acetyl-CoA carboxylase (ACC). VOW also decreased adipogenic-associated protein expressions such as the peroxisome proliferator-activated receptor-γ (PPAR-γ), CCAAT/enhancer-binding proteins α (C/EBP α), sterol regulatory element binding protein-1c (SREBP-1c), and fatty acid synthase (FAS). The enhanced effect of VOW was abolished by the knockdown of AMPK with siRNA. The inhibitory effect of VOW on differentiation depended on the treatment period, even though VOW treatment downregulated the C/EBP β expression at the early phase of differentiation. VOW dramatically reduced activation of AMPK, thereby downregulating adipogenic-associated proteins. Furthermore, the butanol fraction (BtOH) of VOW showed the most powerful effect of VOW dose-dependently reduced lipid accumulation by suppressing the phosphorylation of AMPK. Consistent with inhibited lipid accumulation in vitro, VOW reduced body weight and white adipose tissue weight in the HFHSD-induced obese animal model. Conclusions: Overall, our study suggested that the anti-adipogenesis effect of VOW and its BtOH fraction involved the activation of AMPK.

## 1. Introduction

Obesity has become the toughest challenge in many countries. Obesity has been established as a risk factor for many serious chronic diseases, such as type 2 diabetes, cardiovascular diseases, arthritis, and cancers [1,2,3]. Adipose tissue exhibits a remarkable capacity to undergo hypertrophy and hyperplasia. The processes governing the size and number of adipocytes determine adipose tissue mass [4,5,6]. An increase in differentiation followed by lipogenesis increases the number and the size of adipocytes, whereas the fall in accumulated fat due to lipolysis and apoptosis reduces the mass of adipose tissue [4,6]. Decrease in the mass of adipose tissue reduces the risk of chronic disease [7,8]. There is a lot of research being conducted on the adverse effects of and solutions for obesity [9,10], but considering its high prevalence worldwide and potential for serious complications [11], research on ways to solve obesity without side effects should continue.

Except for exercise and dietary modifications, available approaches for managing obesity are limited and mainly include pharmacotherapeutic medicine and bariatric surgery [12]. Orlistat, a prescription weight loss medication, has been associated with adverse effects, including kidney and liver failure [13,14]. The serious adverse effect of anti-obesity drugs brings many natural food materials to the treatment of obesity and obesity-related metabolic disorders [12,15]. Although many studies have elucidated natural food products with a broad range of biological activities, finding beneficial natural agents with anti-obesity effects remains a challenge.

*Vaccinium oldhamii Miquel* (*V. oldhamii*), a native Korean plant, is a deciduous shrub that belongs to Ericaceae plant family. It has been used to treat vomiting, diarrhea, gonorrhea, eruption, and inflammation [16]. *V. oldhamii* leaves exert a protective role against nitric oxide (NO) production in lipopolysaccharide (LPS)-stimulated RAW264.7 cells [17]. Furthermore, *V. oldhamii* inhibits α-amylase and acetyl cholinesterase [16,18]. The fruit of *V. oldhamii* has higher antioxidant activity [19] than that of blueberries [20]. Moreover, the anthocyanin and polyphenol contents in the fruit of *V. oldhamii* are higher than those in southern and northern highbush blueberries [16]. Therefore, *V. oldhamii* is an important resource for the development of new blueberry cultivars [19].

In this study, we investigated the inhibitory effects of *V. oldhamii* fruit water extract (VOW) on the lipid accumulation in 3T3-L1 cells and a high-fat and high-sucrose diet (HFHSD)-induced obese animal model. We hypothesized that the mechanism of the anti-obesity effect of VOW is AMPK activation. First, we confirmed whether VOW and its active fraction, VOW-BtOH, phosphorylated AMPK in cell and animal models. Next, we showed that the anti-obesity effect of VOW was abolished when AMPK was knocked out using AMPK siRNA. In addition, we identified the butanol fraction (BtOH) as the most bioactive solvent fraction and evaluated its impact on lipid accumulation.

## 2. Materials and Methods

### 2.1. Extract and Fraction Preparation

*V. oldhamii* fruit was bought from Yeonggwang-gun (Republic of Korea). The fruit was then washed with distilled water, freeze-dried, and powdered. Subsequently, 100 g of powder was soaked in distilled water at a ratio of 1:20 and extracted at room temperature for two days. The extract was filtered using qualitative filter paper (90 mm, Hyundai Micro, Seoul, Republic of Korea) and vacuum-concentrated at 40 °C using a vacuum concentrator (Vacuum Pump V-100, Buchi, Gyeonggi-do, Republic of Korea; Rotary Evaporator *N*-1000, Eyela, Tokyo, Japan). Subsequently, it was freeze-dried (FDTA-4508, Operon, Gyeonggi-do, Republic of Korea) and stored at −20 °C for further experiments. The yield of VOW was 16.9%. Fractionation of the VOW was performed using a sequential solvent partitioning method based on polarity differences. All solvents used in the fractionation were of HPLC grade and obtained from Sigma-Aldrich Co. (St. Louis, MO, USA). Initially, a 1:1 (*v*/*v*) mixture of water and hexane was used, yielding a water-soluble and a hexane-soluble fraction. Further fractionation was carried out by sequentially adding chloroform, ethyl acetate, and butanol. Each fraction was concentrated under reduced pressure and subsequently lyophilized to eliminate residual solvents. The fractions were then concentrated under reduced pressure, freeze-dried, and preserved at −20 °C for experimental use.

### 2.2. Cell Culture

For cell culture, 3T3-L1 preadipocytes were purchased from the American Type Culture Collection (ATCC, Manassas, VA, USA). The cells were cultured in Dulbecco’s modified eagle medium (DMEM; #11995065, Gibco, Thermo Fisher Scientific, Waltham, MA, USA) containing 1% penicillin (Gibco, Carlsbad, CA, USA) and 10% newborn calf serum (FCS, #26010074, Gibco). Cells were maintained at 37 °C and 5% CO_2_. For cell differentiation, cells were induced with DMEM containing 10% fetal bovine serum (FBS, Gibco), 1% penicillin, a mixture of 3-isobutyl-1-methylxanthine (0.5 mM IBMX, #28822-58-4, Sigma-Aldrich), insulin (5 mg/mL, #11070-73-8, Sigma-Aldrich), and dexamethasone (1 μM, #D4902, Sigma-Aldrich) for two days. After 2 days, the medium was removed and replaced with insulin-enriched DMEM containing 10% FBS. Cells were subsequently cultured in DMEM supplemented with 10% FBS for an additional four days [21]. To assess the effects of VOW on lipid accumulation during specific differentiation stages, VOW was administrated exclusively within the designated treatment intervals. VO extracts and fractions were dissolved in medium during differentiation.

### 2.3. Cell Viability

The cytotoxicity of VOW was confirmed using the 3-(4,5-dimethylthiazol-2-yl)-2,5-diphenyltetrazolium bromide (MTT; #298-93-1, Biosesang, Gyeonggi-do, Republic of Korea) assay. Next, 3T3-L1 preadipocytes were seeded in a 96-well plate at a density of 1 × 10^4^ and then treated with VOW for an additional 24 h. Next, MTT solution (2 mg/mL) was added to each well, and they were incubated at 37 °C for 4 h. After media removal, cells were treated with dimethyl sulfoxide (DMSO; #D2650, Sigma-Aldrich) to dissolve the formazan crystals. The absorbance was detected at 540 nm using microplate reader (Thermo Fisher Scientific) [22,23].

### 2.4. Lipid Accumulation

Following 8 days of differentiation induction, 3T3-L1 adipocytes were washed with PBS and fixed with 4% paraformaldehyde for 30 min. Lipid droplets were stained for 1 h using Oil Red O (ORO, #O0625, Sigma-Aldrich) prepared in 60% isopropanol. The stained cells were then imaged using EVOX XL Core microscope (Thermo Fisher Scientific) at 20× magnification. Subsequently, the cells were washed with distilled water and de-stained with 10% isopropanol. Absorbance was measured at 540 nm using microplate reader.

To compare inhibitory effect of lipid accumulation across various extracts of *V. oldhamii* (VO), cells were treated with 200 ug/mL of *V. oldhamii* fruit ethanol extract (VOE), *V. oldhamii* fruit boiling water extract (VOB), or *V. oldhamii* fruit water extract (VOW). After 8 days, cells were fixed with ORO solution, and lipid content was quantified by measuring the absorbance at 540 nm.

### 2.5. Animals

Animal studies were approved by the Mokpo National University of Institutional Animal Care and Use Committee (MNU-IACUC-2020-007). A total of 36 C57BL/6J mice (5-week-old, Central Lab. Animal Inc., Seoul, Republic of Korea) were obtained and housed in standard cages (200 × 260 × 130 mm, polycarbonate, Central Lab) lined with bedding material SAFE 3/4S (Central Lab). Two mice were assigned to each cage. The mice were maintained under controlled conditions of 22 ± 2 °C temperature, 50 ± 10% relative humidity, and a 12 h light/dark cycle, and had free access to diet and water. After adaptation, mice are randomly divided into four groups: normal diet-fed control group (ND, n = 9), high-fat and high-sucrose diet-fed control group (HFHSD, n = 9), HFHSD group treated with 200 mg/kg body weight of VOW (HFHSD + VOW 200, n = 9), and HFHSD group treated with 400 mg/kg body weight of VOW (HFHSD + VOW 400, n = 9). ND (15.8% kcal fat, AIN-93G, D10012g) and HFHSD (45% kcal fat and 23% kcal sucrose, D03021303) were purchased from Research Diets, Inc. (New Brunswick, NJ, USA). The mice were orally administered distilled water (ND and HFHSD) or VOW via oral gavage once a day for 13 weeks. Body weight and food intake were monitored weekly. After 13 weeks, the mice were euthanized via cervical dislocation, and blood was collected through cardiac puncture. Serum was separated via centrifugation. White adipose tissue was collected, weighed, and stored at −70 °C for further analysis.

### 2.6. Blood Biochemical Parameters

Serum triglycerides (TG), total cholesterol (TC), and low-density lipoprotein cholesterol (LDL-C) levels were measured using commercial enzyme kits (Eiken Co., Tokyo, Japan) with an Automated Chemistry Analyzer (Beckman Coulter Inc., Brea, CA, USA).

### 2.7. Western Blot

The 3T3-L1 cells and adipose tissues were homogenized in radioimmune precipitation assay buffer (RIPA buffer; Thermo Fisher Scientific) containing 1% protease and phosphatase inhibitor cocktails. Protein concentrations were determined by the Bradford assay (Sigma-Aldrich) and equal amounts of protein (20 μg each) were separated on 12% SDS-PAGE gels, then transferred onto a nitrocellulose membrane (Millipore Billerica, Billerica, MA, USA). The membrane was blocked for 1 h with 5% nonfat milk in tris-buffered saline with 0.1% Tween 20 (TBS-T) buffer. The membrane were incubated overnight with primary antibodies [AMP-activated protein kinase (AMPK), p-AMPK, peroxisome proliferator-activated receptor-γ (PPAR-γ), fatty acid synthase (FAS), Wn3α, acetyl-CoA Carboxylase (ACC), p-ACC, β-catenin, non-phospho (np)-β-catenin, and CCAAT/enhancer-binding protein β (C/EBP β); Cell signaling Technology, Beverly, MA, USA: C/EBP α, sterol regulatory element binding protein-1c (SREBP-1c), and β-actin; Santa Cruz Biotechnology Inc., Dallas, TX, USA] followed by incubation with horseradish peroxidase-conjugated secondary antibodies [goat anti-rabbit or goat anti-mouse (1:10,000)]. Bands were visualized using a chemiluminescent substrate (IMGE-NEX, San Diego, CA, USA), and protein expression was quantified using a chemiluminescent imaging system (UVP, Upland, CA, USA) and Vision Works analysis software (version 6.8, Analysis Software, Upland, CA, USA).

### 2.8. H&E Staining

Epididymal adipose tissue was fixed in 4% formalin. Fixed tissues were mounted in paraffin, cut into 5 μm sections, and stained. To remove paraffin, immerse adipose tissue in xylene for 5 min, repeating three times. Treat with ethanol at concentrations of 100%, 95%, 90%, 80%, and 70%, 5 min each. Stain with hematoxylin (H-3401, Vector Laboratories, Newark, CA, USA) for 5 min, then wash in 0.3% HCl–alcohol for 1 min, followed by rinsing with water. Wash in 0.3% ammonia water for 2 min and rinse again. Stain with eosin (HT110116, Sigma-Aldrich) for 10 s. Treat with ethanol (70%, 80%, 90%, 95%, 100%) for 3 min each, then immerse in xylene for 3 min, repeating three times. Finally, mount the tissue with mounting medium (H-5501, Vector Laboratories). The sections were assessed under a microscope (EVOS 3000 core, Thermo Fisher Scientific, 40× magnification).

### 2.9. HPLC Quantification of Malvidin-3-O-Galactoside and Chlorogenic Acid

An analytic HPLC system equipped with a 1525 binary pump, a 2707 autosampler, and a 2998 photodiode array detector (Waters, Milford, MA, USA) was used to quantify malvidin-3-*O*-galactoside and chlorogenic acid in the VOW on a Synergi 4 μm Max-RP 80 Å (4.6 × 250 mm, Phenomenex, Torrance, CA, USA) at 40°C. The solvents A and B were composed of water/acetonitrile/formic acid with ratios of 87:3:10 and 40:50:10, respectively, and the linear gradient profile was as follows: solvent B 6% (0 min), 20% (20 min), 40% (35 min), 60% (40 min), 90% (45 min), 90% (50 min). The flow rate was 0.5 mL/min and injection volume were 20 μL. UV spectrum of each LC peak was obtained in the ranges of 210–600 nm. The contents of both compounds were quantified by each calibration curve that recorded using LC peak areas (Y) and concentrations (X) under 520 (malvidin-3-*O*-galactoside) and 320 nm (chlorogenic acid).

### 2.10. Statistical Analysis

One-way analysis of variance (ANOVA) was performed using SPSS (version 28.0). All values are expressed as mean ± standard error (SE). Differences were considered statistically significant at *p* < 0.05.

## 3. Results

### 3.1. VOW Suppresses Lipid Accumulation and the Expression of Adipogenic Proteins in 3T3-L1 Cells

First, we tested three different extraction conditions for *V. oldhamii* fruit: ethanol (VOE), boiling water (VOB), and water (VOW). Lipid accumulation in each extract was determined through ORO staining, and the protein expression of p-AMPK, which has been postulated to be a therapeutic target for obesity and its associated chronic diseases, was measured [24,25]. VOB and VOW reduced lipid accumulation more effectively than VOE without cell toxicity (Figure S1A,B). The increase in AMPK phosphorylation was the highest in the VOW-treated group (Figure S1C). Therefore, we selected VOW as the most powerful extract for the remainder of the study.

**Figure 1:**
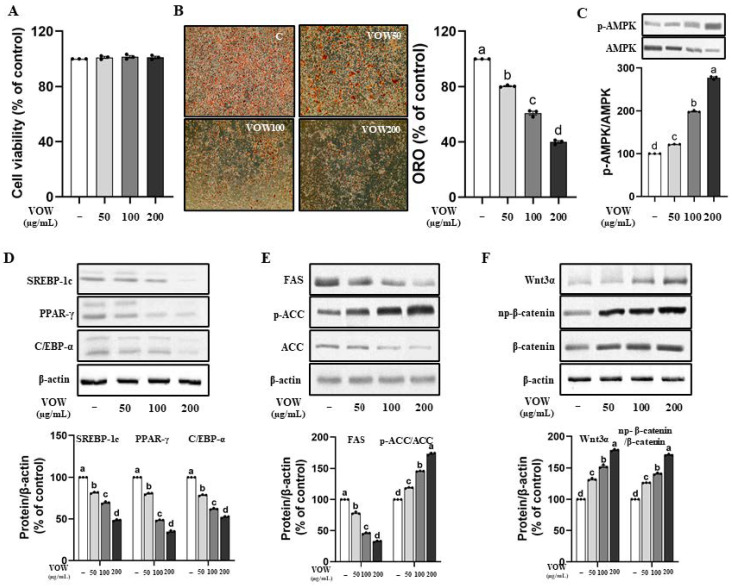
VOW inhibited the lipid accumulation in 3T3-L1 cells. The 3T3-L1 cells were cultured in 10% FBS DMEM medium during the lipid differentiation period. Initially, the cells were treated with MDI-supplemented medium for two days. The medium was then replaced with insulin-only medium for the next two days, followed by switching to DMEM medium every two days thereafter. Cells were differentiated into adipocytes with or without VOW for 8 days. (**A**) Cell viability. (**B**) Oil Red O staining. (**C**) Levels of p-AMPK/AMPK ratio. (**D**) Protein expressions of SREBP-1c, PPAR-γ, and C/EBP-α. (**E**) Protein expressions of FAS and ACC. (**F**) Protein expression pattern of Wnt/β-catenin signaling. All data were generated from three independent biological replicates. Different letters are significantly different by Duncan’s multiple range test (*p* < 0.05).

According to the result of MTT assay shown in Figure 1A, VOW shows no cytotoxicity up to the concentration of 200 µg/mL in 3T3-L1 preadipocytes. VOW markedly inhibited intracellular fat accumulation (Figure 1B) and stimulated AMPK activation (Figure 1C). The expression of transcription factors in differentiation including SREBP-1c, PPAR-γ, and C/EBP α was significantly reduced in a dose-dependent manner by VOW treatment (Figure 1D). In addition, VOW treatment resulted in a downregulation of the expression of two adipogenic enzymes, FAS and ACC, and the upregulation of p-ACC, the inactive form of ACC (Figure 1E). Interestingly, VOW treatment resulted in increased expression of Wnt and np-β-catenin signaling, which is known for its anti-adipogenic properties (Figure 1F).

### 3.2. VOW-Induced Downregulation of Lipid Accumulation Is Associated with the AMPK Activation

**Figure 2:**
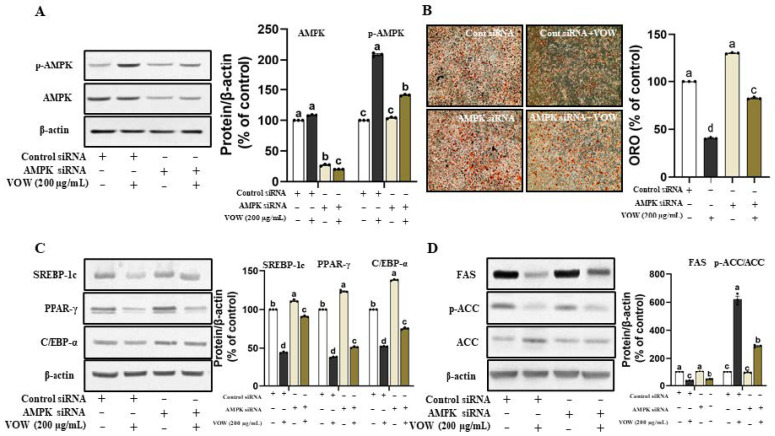
VOW suppressed adipogenesis through AMPK pathway. Cells were transfected with either AMPK α1/α2 siRNA or control siRNA. After 12 h of transfection, cells were differentiated into adipocytes in absence or presence of VOW, following the protocol outlined in Figure 1. (**A**) Protein expressions of AMPK and p-AMPK. (**B**) Oil Red O staining. (**C**) Protein expressions of SREBP-1c, PPAR-γ, and C/EBP-α. (**D**) Protein expressions of FAS and ACC. All data were generated from three independent biological replicates. Different letters are significantly different by Duncan’s multiple range test (*p* < 0.05).

The VOW-mediated increase in AMPK phosphorylation inspired us to examine whether VOW-induced downregulation of lipid accumulation was linked to AMPK activation. The temporary knockdown of AMPK expression (Figure 2A) significantly reduced the inhibitory effect of VOW on the lipid accumulation in 3T3-L1 cells (Figure 2B). Following the lipid accumulation results, AMPK knockdown significantly increased the expression of transcriptional factors of preadipocyte differentiation, such as SREBP-1c, PPAR-γ, and C/EBP-α (Figure 2C). Moreover, the protein expression of FAS and ACC was increased, whereas that of p-ACC, the inactive form of ACC was reduced in VOW-treated groups by temporary knockdown of AMPK (Figure 2D).

### 3.3. The Influence of VOW Depends on Timing and Period of Treatment

**Figure 3:**
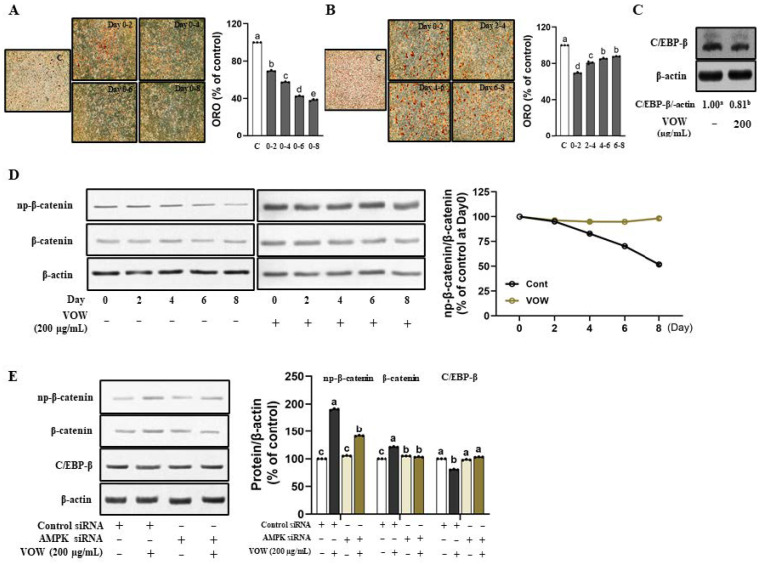
VOW attenuated the early phase of differentiation in 3T3-L1 cells. Cells were differentiated with or without VOW treatment at specific time points. (**A**) Lipid accumulation was evaluated during the lipid formation period (0–8 days) with the additional treatment of VOW (200 µg/mL) at two-day intervals (0–2 days, 0–4 days, 0–6 days, and 0–8 days). (**B**) Lipid accumulation was evaluated during the lipid formation period (0–8 days) with VOW (200 µg/mL) treated for only two days at specific intervals (0–2 days, 2–4 days, 4–6 days, and 6–8 days). (**C**) Protein expression of C/EBP-β. (**D**) Protein expressions pattern of np-β-catenin during the different time point of adipogenesis. (**E**) Expression of β-catenin and C/EBP-β with control siRNA or AMPK siRNA. All data were generated from three independent biological replicates. Different letters are significantly different by Duncan’s multiple range test (*p* < 0.05).

Next, we treated cells with VOW at the concentration of 200 µg/mL along with the differentiation medium for various time intervals in different periods, to determine the key timing and period of treatment at which VOW showed its profound inhibitory effect. According to the ORO results, the longer the treatment period, the greater was the influence of VOW on the preadipocyte differentiation (Figure 3A). When the treatment period was the same, the earlier the treatment time, the greater the influence (Figure 3B). We also found that VOW significantly inhibited the expression of C/EBP-β, a key transcription factor involved in the early stages of differentiation (Figure 3C). np-β-catenin, the negative regulator of adipogenesis, was known to be expressed in the early phase of differentiation and can directly suppress the expression of C/EBP-α and PPAR-γ [18]. Our data demonstrate that VOW not only increased the expression of np-β-catenin in the early stage of differentiation, but also maintained the expression level until the late stage of differentiation (Figure 3D). Moreover, VOW-induced regulation in the expression of np-β-catenin and C/EBP-β expressions was abolished by AMPK knockdown (Figure 3E).

### 3.4. Butanol Fraction of VOW Is the Most Effective Among Various Solvent Fractions in Suppressing Lipid Accumulation in 3T3-L1 Cells

To determine the most effective fraction of VOW, we first assessed cell viability. The 3T3-L1 cells were treated with 200 µg/mL of each VOW fraction, as shown in Supplementary Figure S2A. The viability of ethyl acetate and butanol fraction (VOW-BtOH)-treated cells was reduced, but was still above 85% of control value (Figure S2B). Lipid accumulation determined by ORO showed that VOW-BtOH was the most effective solvent fraction (Figure S2C). Therefore, we investigated the inhibitory effect of VOW-BtOH on lipid accumulation and the expression of adipogenic proteins.

**Figure 4:**
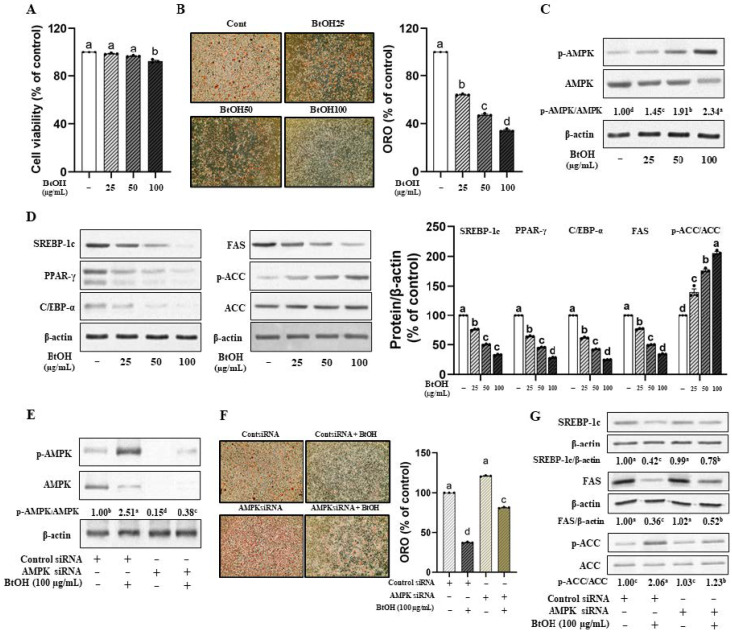
Butanol fraction from VOW downregulated lipid accumulation via AMPK pathway. Cells were transfected with either AMPK α1/α2 siRNA or control siRNA. After 12 h of transfection, cells were differentiated into adipocytes in absence or presence of VOW-BtOH. (**A**) Cell viability. (**B**) Lipid accumulation of BtOH fraction. (**C**) Protein levels of p-AMPK/AMPK ratio. (**D**) Protein expressions of SREBP-1c, PPAR-γ, C/EBP-α, FAS, and ACC. (**E**) Protein expressions of AMPK and p-AMPK. (**F**) Lipid accumulation of BtOH fraction in the absence or presence of AMPK siRNA. (**G**) Protein expression of lipogenesis in the absence or presence of AMPK siRNA. All data were generated from three independent biological replicates. Different letters are significantly different by Duncan’s multiple range test (*p* < 0.05).

The cell viability, as indicated by the IC_50_, showed unaffected at concentrations up to 100 µg/mL VOW-BtOH (Figure 4A). VOW-BtOH treatment reduced lipid accumulation in a dose-dependent manner (Figure 4B) and increased AMPK activation (Figure 4C). As expected, the protein expression of the transcription factors was reduced by VOW-BtOH treatment. Additionally, the levels of lipogenic enzymes including FAS and ACC were reduced (Figure 4D). Consistent with the effect of VOW, the temporary knockdown of AMPK expression (Figure 4E) reversed the suppressive effect of VOW-BtOH on the lipid accumulation in 3T3-L1 cells (Figure 4F). Moreover, AMPK knockdown abolished VOW-BtOH-induced changes in FAS, ACC, and p-ACC protein expressions (Figure 4G).

**Figure 5:**
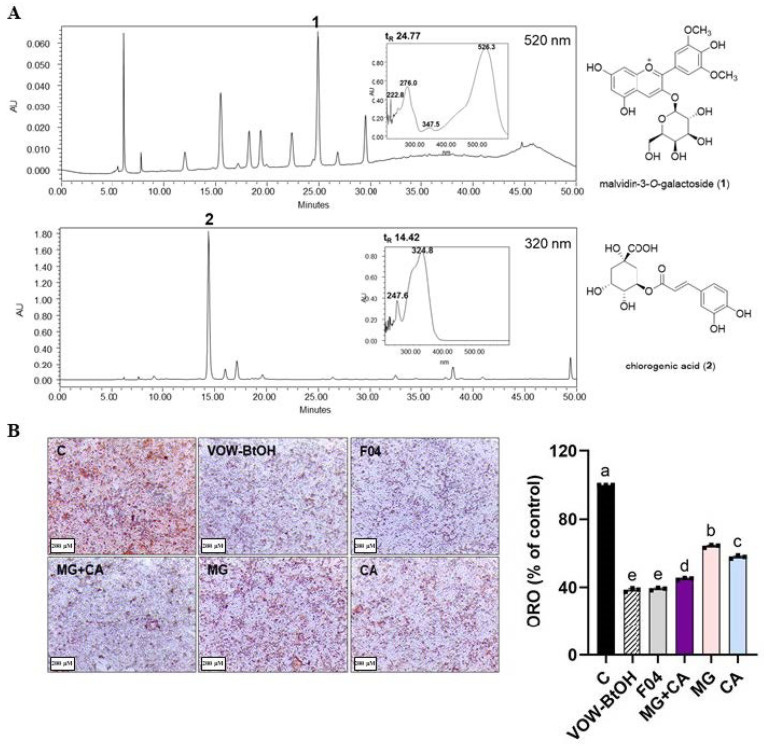
HPLC analysis and lipid accumulation inhibitory effect of each fraction and active compound of VOW. (**A**) HPLC profiles of the VOW, malvidin-3-*O*-galactoside (upper panel) and chlorogenic acid (lower panel). (**B**) Oil Red O staining. ORO data were generated from three independent biological replicates. Different letters are significantly different by Duncan’s multiple range test (*p* < 0.05).

Based on the inhibitory effect on lipid accumulation, we identified VOW-BtOH as the active solvent fraction (Figure 5A) and examined its underlying mechanism of action. Consistent with VOW, VOW-BtOH exerted anti-adipogenic effects via AMPK-mediated regulation. Furthermore, we determined the most active subfraction (SF04) from VOW-BtOH and identified two presumed active compounds, malvidin 3-O-galatoside (MG) and chlorogenic acid (CA) (Figure 5A). The inhibitory effect of 100 µg/mL VOW-BtOH and the amount of subfraction (SF04), MG, CA, and MG + CA contained therein was compared and VOW-BtOH treatment showed the most potent inhibitory effect on lipid accumulation (Figure 5B).

### 3.5. VOW Suppresses High-Fat and High-Sucrose Diet (HFHSD)-Induced Effects in Body Weight, Adipose Tissue Weight, and Serum Lipids

**Figure 6:**
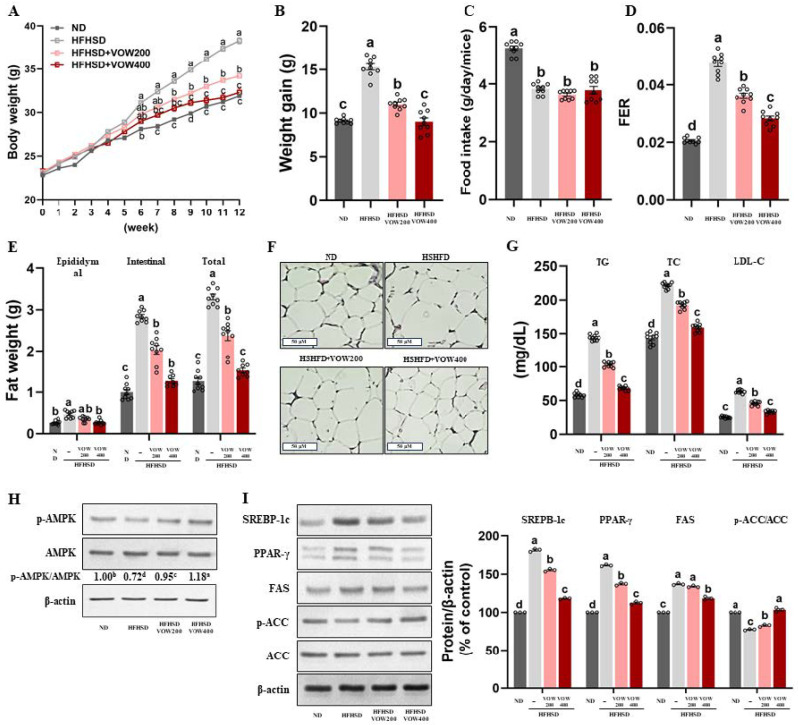
VOW inhibited body and fat weight in high-fat high-sucrose diet-induced obesity. (**A**) Body weight for 12 weeks. (**B**) Weight gain. (**C**) Food intake. (**D**) FER. (**E**) Fat weight. (**F**) H&E staining in white adipose tissue. (**G**) Serum lipid metabolism. (**H**) Levels of p-AMPK/AMPK ratio. (**I**) Protein pattern of lipogenesis in white adipose tissue. Immunoblotting data were generated from three independent biological replicates. Different letters are significantly different by Duncan’s multiple range test (*p* < 0.05).

To assess the effect of VOW on weight loss, we measured body and fat weights in an HFHSD-induced obese animal model. As shown in Figure 6A, VOW significantly reduced body weight compared to the control group. Specifically, 400 mg/kg/day VOW suppressed weight gains to the levels observed in the normal control group (Figure 6B). Given that absence of difference in food intake among HFHSD groups (Figure 6C), VOW reduced food efficacy ratio (Figure 6D) through mechanisms independent of food intake reduction. Consistent with the result of body weight, VOW suppressed the total, epididymal, and intestinal adipose tissue weights in HFHSD-fed animals (Figure 6E). H&E staining also showed a VOW-induced decrease in the adipose tissue cell size (Figure 6F). Furthermore, VOW resulted in a decrease in the serum levels of triglycerides, total cholesterol, and LDL-cholesterol (Figure 6G).

Next, we tested whether VOW treatment was involved in the regulation of adipogenic transcription factors via AMPK activation. As expected, VOW effectively stimulated AMPK activation, thereby increasing p-AMPK/AMPK levels (Figure 6H). The expression of transcription factors in differentiation including SREBP-1c and PPAR-γ in white adipose tissues was remarkably reduced in a dose-dependent manner by VOW treatment (Figure 6I). VOW treatment downregulated the expression of two adipogenic enzymes, FAS and ACC, but upregulated p-ACC, the inactive form of ACC (Figure 6I).

## 4. Discussion

Since obesity and related diseases are considered a worldwide health problem, research on natural agents with anti-obesity effects is ongoing. In this study, we prepared a water extract of *V. oldhamii* fruit, which has higher anti-oxidant activity than blueberries [20], and examined its anti-obesity effect and associated mechanism of action in both cell and animal models.

We investigated the inhibitory effect of VOW on 3T3-L1 cell differentiation. ORO staining reveals that lipid accumulation during differentiation was dose-dependently reduced by VOW treatment. Adipogenesis is controlled by a transcriptional cascade that harmonizes changes in the expression of specific adipocyte genes. AMPK activation is associated with the inhibition of adipogenesis [18,19,20]. Phosphorylated AMPK inactivates ACC and decreases the cellular concentration of malonyl-CoA, resulting in decreased lipogenesis [21]. According to our results, VOW significantly suppressed adipogenesis by activating AMPK, thereby phosphorylating its downstream protein, ACC to p-ACC. Consistent with these results, the temporal knockdown of AMPK shows that AMPK is the target protein of VOW. Furthermore, the protein expression of C/EBP α and PPAR-γ, two master transcription factors for terminal differentiation [25,26], is significantly downregulated by VOW treatment.

When cells were treated with VOW for two different days during differentiation (from day 0 to 2, day 2 to 4, day 4 to 6, or day 6 to 8), treatment during the early stage of differentiation (days 0–2) resulted in the greatest reduction in lipid accumulation. The protein expression of C/EBP-β, the critical transcription factors for early phase [23] and the upstream regulator of C/EBP-α and PPAR-γ [18], showed a decrease in the VOW-treated group, which suggests that VOW has an influence on the upstream part of the adipogenesis in the early phase.

Canonical Wnt/β-catenin signaling is a key source of stem cell proliferation and differentiation [27,28,29,30]. Wnt/β-catenin signaling is the negative regulator of adipogenesis [28,29,30,31]. The activation of Wnt signaling via LiCl or CHIR 99021, both glycogen synthase kinase 3 (GSK3) inhibitors, led to an increase in β-catenin expression, consequently resulting in a reduction in C/EBP-α and PPAR-γ expression [32,33]. Most importantly, C/EBP-α and PPAR-γ serve as crucial transcriptional factors during the late stage of proliferation, facilitating mature adipocytes [28,34]. VOW dose-dependently increased the expression of Wnt3a and np-β-catenin, which can translocate from the cytoplasm into the nucleus and directly suppresses the expression of C/EBP-α and PPAR-γ (Figure 1). The most notable result was that (np)-β-catenin expression in the control group decreased after day 2, whereas VOW treatment maintained expression throughout the differentiation period (Figure 3). It is speculated that the sustained increase in np-β-catenin expression may have contributed to the continuous induction of C/EBP-α and PPAR-γ in the late differentiation period.

Several studies have suggested that activation of AMPK is associated with β-catenin-dependent Wnt signaling [35]. When AMPK is activated by metformin, it can sequester β-catenin within cytoplasm, thereby augmenting proteasomal degradation of β-catenin rather than promoting its nuclear translocation [36]. In this study, VOW inhibits the early phase of differentiation by increasing the activation of AMPK, the upstream of C/EBP-β and np-β-catenin, thereby inhibiting both the early phase of differentiation and the expression of the transcriptional factors in late stage of differentiation. The inhibitory effect of VOW on the expression of C/EBP-α and PPAR-γ is further increased by maintaining the expression level of np-β-catenin until the late stage of differentiation. Therefore, the longer the treatment period, the stronger the inhibitory effect on lipid accumulation.

Our results suggest that the anti-obesity effects of VOW-BtOH are likely driven by the synergistic interaction of multiple bioactive compounds, including MG and CA, rather than a single active compound. Previous studies have shown that MG reduces lipid accumulation in human hepatocytes by regulating TFEB-mediated lysosomal function and enhancing the redox state through Nrf2/ARE activation [37], while CA mitigates lipid accumulation in oleic acid-induced HepG2 cells [38], high-fat diet-fed zebrafish [38], and 3T3-L1 cells [39]. Consistent with these findings, our study identifies SF04, which contains both MG and CA, as the most bioactive subfraction, supporting the idea that the observed anti-obesity effects of VOW-BtOH result from the synergistic activity of multiple compounds. However, a limitation of our study is that the exact mechanisms underlying the interaction between these compounds are not fully understood. Additionally, the effects observed may be due to the combined action of several components in the natural product, rather than a single compound, highlighting the need for further research to clarify the molecular pathways involved.

## 5. Conclusions

In conclusion, our study provides an initial step towards in investigating the anti-obesity effect of VOW, in vitro and in vivo. Its mechanism is associated with AMPK-mediated control of adipogenesis. Furthermore, we suggested C/EBP-β and β-catenin as another target proteins to suppress the adipogenesis in the early phase of differentiation. In addition to the cell-based assay results, VOW showed a profound anti-obesity effect in the HFHSD-fed animal model. VOW administration significantly reduced both body fat accumulation and weight gain. Considering that the primary focus of obesity treatment is to reduce the risk of obesity-related diseases including cardiovascular disease (CVD), VOW is a promising agent for treating obesity because it can improve obesity-related health problems, including dyslipidemia.

## Data Availability

The original contributions presented in this study are included in the article/Supplementary Materials. Further inquiries can be directed to the corresponding author.

## Supplementary Materials

The following supporting information can be downloaded at: https://www.mdpi.com/article/10.3390/nu17081346/s1, Figure S1. Effect of various extracts of *Vaccinium oldhamii* on lipid accumulation. Figure S2. Effect of VOW fractionation on lipid accumulation. Table S1. List of primary antibodies.
