# Supplementary material for: Vaccinium oldhamii Fruit Inhibits Lipid Accumulation in 3T3-L1 Cells and Diet-Induced Obese Animals
Source: Nutrients. 2025 Apr 14;17(8):1346. doi: 10.3390/nu17081346 (PMC12030422; doi:10.3390/nu17081346)
Supplement: Supplementary file 1 [file nutrients-17-01346-s001.zip › nutrients-3558441-supplementary.pdf]

Supplemental Information

Vaccinium oldhamii fruits inhibits lipid accumulation in 3T3-L1 cells and diet-induced obese animals

Young-Hyun Lee[1†], Mikyoung You[1†], Hyeon-A Kim[1*]

[1]Department of Food and Nutrition, Mokpo National University, Jeollanam-do, 58554, Republic of Korea

2Convergence Center for Green Anti-Aging Research, Mokpo National University, Muan-Gun, 58554, Republic of Korea

† These authors contributed equally to this work.

Inventory of Supplemental Information

Supplemental Figures

Figure S1. Effect of various extracts of *Vaccinium aldhamii* on lipid accumulation.

Figure S2. Effect of VOW fractionation on lipid accumulation.

Supplemental Table

Table S1. List of primary antibodies.

Supply Figure 1

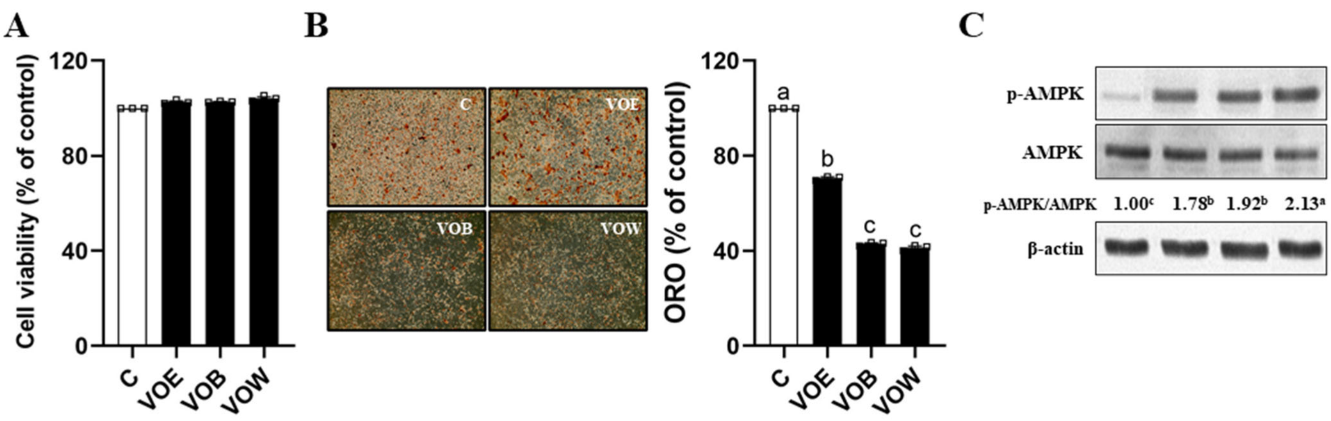

Figure S1. Effect of various extracts of Vaccinium aldhamii on lipid accumulation.
A) Cell viability. B) Oil Red O staining. C) Expression of AMPK and p-AMPK. *Different letters are significantly different by Duncan's multiple range test (P < 0.05).*

Supply Figure 2

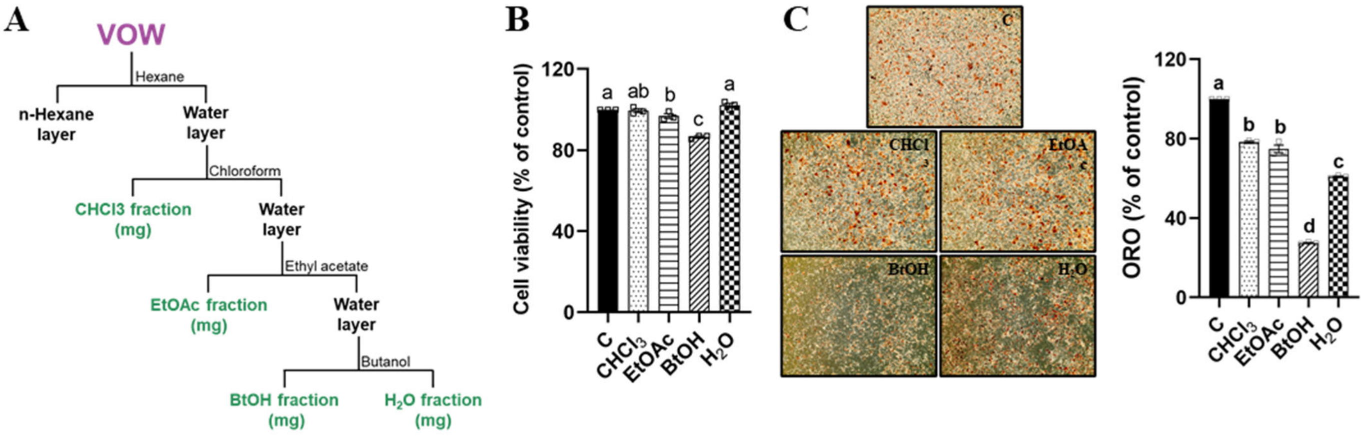

Figure S2. Effect of VOW fractionation on lipid accumulation.

A) Method of fractionation of VOW. B) Cell viability. C) Oil Red O staining. *Different letters are significantly different by Duncan's multiple range test (P < 0.05).*

Table S1. List of primary antibodies

Antibody	Host	Dilution	Company	Catalog no.
SREBP-1c	Rabbit	1:1000	Santa Cruz Biotechnology	SC-366
FAS	Rabbit	1:1000	Cell Signaling	3180

p-ACC	Rabbit	1:1000	Cell Signaling	3661
ACC	Rabbit	1:1000	Cell Signaling	3662
p-AMPK	Rabbit	1:1000	Cell Signaling	2535
AMPK	Rabbit	1:1000	Cell Signaling	2532
Wnt3α	Rabbit	1:1000	Cell Signaling	2721
β-catenin	Rabbit	1:1000	Cell Signaling	9562
np-β-catenin	Rabbit	1:1000	Cell Signaling	8814
C/EBP-α	Rabbit	1:1000	Santa Cruz Biotechnology	SC-61
C/EBP-β	Rabbit	1:1000	Cell Signaling	3087
β-actin	Mouse	1:1000	Santa Cruz Biotechnology	sc-47778
